# Xanomeline/trospium chloride: a new therapeutic strategy in schizophrenia

**DOI:** 10.1192/j.eurpsy.2026.11784

**Published:** 2026-07-31

**Authors:** H. Becerra Darriba, P. E. Paredes Carreño, S. Oliver Hernández

**Affiliations:** Servicio Navarro de Salud-Osasunbidea , Tudela, Spain

## Abstract

**Introduction:**

Schizophrenia is a severe, chronic psychiatric disorder marked by positive, negative, and cognitive symptoms and associated with substantial disability. While D2 dopamine receptor–blocking antipsychotics effectively reduce positive symptoms, many patients remain partially responsive or treatment‑resistant, contributing to poor adherence and relapse. Xanomeline is an oral M1/M4 muscarinic agonist without direct D2 blockade, which is combined with trospium chloride, a peripherally restricted muscarinic antagonist, to mitigate peripheral cholinergic side effects while preserving central efficacy.

**Objectives:**

To synthesize randomized controlled trials and recent meta-analyses on the efficacy, safety, and comparative effectiveness of xanomeline/trospium chloride for schizophrenia.

**Methods:**

A systematic search of PubMed, Embase, Scopus, Web of Science databases was conducted using the following search terms: “*muscarinic receptors* AND *schizophrenia*”, “*M1/M4 agonist*”, “*xanomeline*, “*trospium chloride*”. Inclusion criteria: original peer reviewed studies, targeting M1–M5 mAChRs. Exclusion criteria: nonspecific cholinergic agents, case reports. The review was conducted in accordance with PRISMA guidelines and articles published in the last 5 years were selected.

**Results:**

We consolidated evidence from phase 2–3 RCTs and quantitative syntheses, following PRISMA-aligned methods, focusing on PANSS outcomes, responder rates, and safety. Four studies (three RCTs and one post hoc; n=690) were included in recent meta-analytic syntheses. Xanomeline/trospium chloride significantly reduced PANSS total scores versus placebo (MD −13.77; 95% CI −22.33 to −5.20) and improved positive and negative subscales, and it more than doubled the likelihood of achieving a ≥30% PANSS reduction (RR 2.15; 95% CI 1.64–2.84). Placebo-controlled RCTs (EMERGENT-1/2) consistently demonstrated clinically meaningful improvements across primary and secondary endpoints. Xanomeline increased cholinergic gastrointestinal events but no signal for akathisia, weight gain, or metabolic changes, aligning trial and meta-analytic findings. In meta-analysis, xanomeline’s short-term efficacy on total, positive, and negative symptoms was comparable to aripiprazole, risperidone, and olanzapine, with less short-term weight gain than risperidone and olanzapine. The FDA has approved xanomeline–trospium for the treatment of schizophrenia in adults, underscoring clinical relevance.

**Conclusions:**

Xanomeline–trospium validates muscarinic receptor agonism as an effective, non-dopaminergic antipsychotic strategy that improves core schizophrenia symptoms while avoiding key liabilities of D2-blocking agents (extrapyramidal effects and weight gain), albeit with increased cholinergic gastrointestinal effects. Priorities include defining long-term efficacy, relapse prevention, adherence, and neurological safety (tardive dyskinesia), where current evidence remains limited.

**Disclosure of Interest:**

None Declared

